# Rural and urban differences in metabolic profiles in a Cameroonian population

**DOI:** 10.4314/pamj.v10i0.72204

**Published:** 2011-09-02

**Authors:** Clarisse Noël Ayina Ayina Lissock, Eugène Sobngwi, Eliane Ngassam, Laurent Serge Ngoa Etoundi

**Affiliations:** 1 Laboratory of animal Physiology, Higher Teacher's Training College, University of Yaoundé I, P.O. Box: 47 Yaoundé, Cameroon; 2 Department of Internal Medicine and Specialties, Faculty of Medicine and Biomedical Sciences, University of Yaoundé I, Cameroon

**Keywords:** Adults, anthropometry, lipid profile, blood glucose, blood pressure, diabetes, urban, rural, Cameroon

## Abstract

**Introduction:**

The difference between modern lifestyle in urban areas and the traditional way of life in rural areas may affect the population's health in developing countries proportionally. In this study, we sought to describe and compare the metabolic (fasting blood sugar and lipid profile) profile in an urban and rural sample of a Cameroonian population, and study the association to anthropometric risk factors of obesity.

**Methods:**

332 urban and 120 rural men and women originating from the Sanaga Maritime Department and living in the Littoral Region in Cameroon voluntarily participated in this study. In all participants, measurement of height, weight, waist circumference, hip circumference, blood pressure systolic (SBP) and blood pressure diastolic (DBP), resting heart rate (RHR), blood glucose and lipids was carried out using standard methods. Total body fat (BF%) was measured using bio-impedancemetry. Body mass index (BMI) and waist to hip ratio (WHR) were calculated. Low Density Lipoprotein-cholesterol (LDL-c) concentrations were calculated using the Friedwald formula. World Health Organization criteria were used to define high and low levels of blood pressure, metabolic and anthropometric factors.

**Results:**

The highest blood pressure values were found in rural men. Concerning resting heart rate, only the youngest women's age group showed a significant difference between urban and rural areas (79 ± 14 bpm vs 88 ± 12 bpm, p = 0.04) respectively. As opposed to the general tendency in our population, blood glucose was higher in rural men and women compared to their urban counterparts in the older age group (6.00 ± 2.56 mmol/L vs 5.72 ± 2.72 mmol/L, p = 0.030; 5.77 ± 3.72 vs 5.08 ± 0.60, p = 0,887 respectively). Triglycerides (TG) were significantly higher in urban than rural men (1.23 ± 0.39 mmol/L vs 1.17 ± 0.64 mmol/L, p = 0.017). High Density Lipoprotein-cholesterol (HDL-c) levels were higher in rural compared to urban men (2.60 ± 0.10 35mmol/L vs 1.97 ± 1.14 mmol/L, p<0.001 respectively). However, total Cholesterol (TC) and LDL-c were significantly higher in urban than in rural men (p<0.001 and p = 0.005) and women (p<0.001 respectively. Diabetes’ rate in this population was 6.6%. This rate was higher in the rural (8.3%) than in the urban area (6.0%). Age and RHR were significantly higher in diabetic women than in non-diabetics (p = 0.007; p = 0.032 respectively). In a multiple regression, age was an independent predictor of SBP, DBP and RHR in the entire population. Age predicted blood glucose in rural women only. BMI, WC and BF% were independent predictors of RHR in rural population, especially in men. WC and BF% predicted DBP in rural men only. Anthropometric parameters did not predict the lipid profile.

**Conclusion:**

Lipid profile was less atherogenic in rural than in urban area. The rural population was older than the urban one. Blood pressure and blood glucose were positively associated to age in men and women respectively; this could explain the higher prevalence of diabetes in rural than in urban area. The association of these metabolic variables to obesity indices is more frequent and important in urban than in rural area.

## Introduction

Storage of excess calories as fat must ultimately result from a net positive energy balance over time. Many physiological systems (endocrine, gastrointestinal, cardiovascular, central and peripheral nervous systems) affect the energy intake, energy expenditure and partitioning of energy stores as fat, carbohydrate, and protein. Small changes in any of these functions can, over time, affect the body composition and metabolism, resulting in excess general and abdominal adiposity, deposition of ectopic fat and increased BMI, blood pressure dyslipidemia and type 2 diabetes [1,2].

Many other factors have been associated to the occurrence of cardiovascular diseases. A study done in 51 countries, most of which were developing countries, in the World Health Survey (2002–2003) showed that due to physical inactivity, about 15% of men and 20% of women were at risk for chronic diseases [3]. However, the responsibility of genetics in the occurrence of obesity and related disorders has been and remain a very interesting subject of investigation [4].

Rural-urban differences in metabolic profiles are noted in most developing countries [5]. These differences may be due to demographic transition (shift to low fertility, low mortality, and higher life expectancy) and epidemiologic transition (from widely prevalent infectious diseases to a pattern of high prevalence of chronic life style related non communicable diseases (NCD)), as these countries become more resourceful economically (socioeconomic transition, shift of people from the rural to the urban area). These factors are responsible for significant changes in dietary and physical activity patterns (nutrition and lifestyle transitions, and stress) leading to an increased burden of cardiovascular diseases (CVD) [2,6].

Africa, jointly with many developing regions, is currently undergoing one of the most rapid demographic and epidemiologic transitions in the world, and as consequence, the shift in the pattern of the NCDs is occurring at a faster rate than it did in the industrialized regions of the world half a century ago [2,7,8]. The number of people with diabetes is expected to double (from 2000 to 2030) in three of the six developing regions, including the Middle East and North Africa, South Asia, and Sub-Saharan Africa [9]. The prevalence of hypertension, diabetes and recently, metabolic syndrome reported for the sub-Saharan population is very alarming [10–13].

For many reasons, urbanization and modernization occur differently in the Sub Saharan African regions; the rural-to-urban migration that exposes migrants to urbanized diets and lifestyles is very important. Changes in occupations, the advent of new technologies, and the rapid pace of urban life have resulted in more sedentary work and less energy expenditure [14,15]. People have diets rich in saturated fats, cholesterol, and refined carbohydrates and poor in polyunsaturated fatty acids and fibre. These finding habits are always associated with markedly sedentary lifestyles and increased stress. This pattern may lead to a pathological metabolic profile. This study was thus carried out in the Littoral Region in Cameroon to describe and compare the metabolic profiles of men and women in urban and rural areas, in diabetics and non-diabetics.

## Methods

### Settings, population and study design

This study was carried out in urban and rural areas in the Littoral Region with Edea (capital of the Sanaga maritime department) being the urban area and Ngambe villages (Songmbengue, Putkak, Ngombee, Tekibo'o) taken as rural area. The Littoral is one of Cameroon's ten administrative regions. It is situated in the western part of Cameroon near the seaside, and is characterized by the presence of a harbour which is one of the most important in Sub Saharan African. The Littoral Region comprises four Division amongst which the Sanaga Maritime Division. The recent census reported 2 865 795 inhabitants in the region in 2010, 14.8% of the Cameroon's population [16]. The population is young (24 years as mean) as is the case in the rest of country. The Littoral Region has the highest rate of urbanization (92.6 %), and the least populated rural areas of Cameroon (7.39%) because of the marked rural-urban migration. The rural zone of Ngambe harbours 3637 inhabitants, which account for 58.57% of the district, 2.24% of the divisional and 0.14% of the regional population. The urban area is mainly made of workers of the public and private sectors, traders and students. In the rural area, most of the inhabitants are farmers [16].

This study was carried out on the natives of the Sanaga Maritime Division. Their traditional diet is characterised by highly greasy traditional foods, with an important quantity of palm oil or palm seed juice, and/or groundnuts in most of their meals. Because of the important rural-urban migration rate in the Littoral Region and since the quality of life and the environment greatly impact the occurrence of diseases, we found interesting to evaluate the metabolic impact of the Sanaga Maritime population's way of life.

As a result of the absence of complete population registers, the sampling was based on the estimated prevalence of obesity of the Littoral Region as reported by the CamBod study in 2004 as being 6.06% in the urban area. We fixed the prevalence in the rural area at 5%, since the exact prevalence was not found, and we considered those reported by Mbanya in rural Cameroon in 1999 as 2 and 3% for men and women respectively [17,18]. We expected 110 and 255 subjects in rural and urban areas respectively. Adult men and women aged 18 and above were invited to participate in this study. Pregnant women and subjects with severe illnesses or physical handicap that could not permit anthropometric measurements were not included in the study. Participants were randomly recruited by announcements in public places, churches and meeting.

Data collection took place at the nearest public health centre. The eligible volunteers who observed a fasting period of at least 10 hours were provided detailed information about the study verbally and on paper. All participants gave their written informed consent before they were enrolled in the study. Blood and clinical data were collected by nurses; the anthropometric data were collected by the researchers and their assistants. All the personnel were trained for the purpose of the study. The collected blood was centrifuged and the serums were stored the same day at −20°C for transportation to the biochemical laboratory of the Yaoundé University Hospital Centre for lipid analysis in a tightly closed ice container.

### Anthropometric measurements

Body weight was measured to the nearest 0.1 kg. Body height was measured to the nearest 0.5 cm using a portable locally manufactured stadiometer. Subjects stood upright on a flat surface without shoes, with the back of the heels and the occiput touching the stadiometer. BMI was calculated as weight (kg) divided by the square of height (m^2^). Waist and hip circumferences were measured in cm using a measuring tape. Waist circumference (WC) was measured midway between the lower rib margin and anterior iliac crest, whereas hip circumference (HC) was measured at the outermost points of the greater trochanters [20]. The waist to hip ratio (WHR) was the ratio calculated using these two circumferences. Percentage body fat (BF%) and total fat mass were measured by bioelectric impedance analysis (OMRON BF 302, OMRON Matsusaka Co., Ltd. Japan). BMI, WC, WHR categories were determined according to WHO, with overweight defined as BMI=25–29.9 kg/m^2^; WC=80-87.9 cm in women and WC=94–101.9cm in men; WHR=0.80-0.84 in women and WHR=0.90-0.99 in men; and obesity as BMI ≥30 kg/m2; WC ≥ 88 cm in women and WC≥102cm in men; WHR ≥ 0.85 in women and WHR ≥ 1.0 an men [19]. For BF%, over weight was defined as BF=28–32% in women and BF=21–28% in men, and obesity as BF>32% in women and BF>28% in men. All anthropometric measurements were performed by the same investigator.

### Blood pressure and resting heart rate measurements

Participants had to be seated for at least 5 min before the measurement of diastolic blood pressure (DBP), systolic blood pressures (SBP) and resting heart rate (RHR). Those parameters were recorded twice on the left arm with five minutes interval between the two measurements, using “Predicor” electronic sphygmomanometer. The average of the two measures was used. To define high levels of blood pressure the recent criteria recommended by the WHO were used; hypertension: SBP ≥ 140 mm Hg and/or DBP ≥ 90 mm Hg, or use of blood pressure lowering drugs.

### Laboratory investigations

Blood glucose was measured between 7 and 10 am after a minimum of 10 hs overnight fast using a One Touch Ultra glucose meter (LifeScan Scotland) on total fresh capillary blood samples. The glucose meter was calibrated each day. Blood glucose level was high when ≥ 5.6mmo/l and Diabetes was defined following the recent criteria as blood glucose level ≥ 7mmol/L.

The blood was withdrawn from the antecubital vein after a minimum of 10 hs fast and was analysed within 1 week. Levels of total cholesterol, HDL-c and TG were determined using a spectrophotometer according to the SGMitalia kit material referenced (Cholesterol 30084 Rev. 0 of 2002-12, Triglycerides LR 30507 Rev. 0 of 2002-12 and HDL Cholesterol PEG 6000 LR30528 Rev. 1 of 2009-12 respectively). The very-low-density-lipoprotein (VLDL) cholesterol was estimated by dividing TG by five, and LDL-c was indirectly calculated by subtracting HDL and VLDL from total cholesterol [21,22]. Serum standards used for calibration were given by the manufacturer. High Total cholesterol, triglycerides, high LDL-c and low HDL c were defined using the following criteria: Total cholesterol ≥5.2 mmol/L; triglycerides ≥1.70 mmol/L; LDL-c ≥2.5 mmo/L; HDL-c ≤1.03 mmol/L for men and HDL-c≤1.29 mmol/L for women.

### Statistical analysis

Analyses were performed using SPSS version 12.0. Means ± SD of all variables were calculated for urban and rural men and women. Comparison across groups was done using the non-parametric Mann-Whitney U test. Chi square test was used to compare the risk factor rates between study areas. Correlations between variables was analysed using the non-parametric Spearman Rank Order Test. Linear regression analysis was used to determine independent predictors of the different metabolic parameters.

### Ethical considerations

The National Ethical Committee of the Ministry of Public Health of Cameroon approved the study. Permission to carry out this study was obtained from local authorities.

## Results

### Characteristics of the population

A total of 453 adults (120 rural men and women, and 332 urban men and women) aged 18 years and above participated in this study. The participation rate was higher than expected. Although 10% of the blood measurements (Lipid profile) not considered because of haemolysis, the sample is still important.

There were 287 women and 165 men in this study. The middle age group (40–55 years) gave the higher response rate (38.9%). Participants’ mean age was higher in the rural than the urban area both in women (51.77 ± 16.34 yearsvs 48.16 ± 13.59 years, p=0.112) and men (52.98 ± 16.79 yearsvs 50.65 ± 15.08 years, p=0.303) but the difference was not significant (Table 1). 

**Table 1:** General characteristics of the study population in urban and rural areas

	Men			Women		
	
	Urban	Rural	P	Urban	Rural	P
n	106	59		226	61	
**Age (years)**	50.65 ± 15.08	52.98 ± 16.79	0.303(NS)	48.16 ± 13.59	51.77 ± 16.34	0.112(NS)
**Height (cm)**	1.70 ± 0.07	1.67 ± 0.07	0.006	1.62 ± 0.07	1.58 ± 0.08	<0.001
**Weight (kg)**	77.00 ± 15.64	65.81 ± 13.54	<0.001	77.24 ± 15.99	61.34 ± 16.26	<0.001
**BMI (kg/cm^2^)**	26.43 ± 4.72	23.63 ± 4.33	<0.001	29.60 ± 5.86	24.60 ± 5.96	<0.001
**WC (cm)**	91.74 ± 12.28	85.58 ± 10.98	0.001	96.28 ± 12.56	85.97 ± 15.03	<0.001
**WHR (cm)**	0.91 ± 0.07	0.90 ± 0.07	0.690(NS)	0.88 ± 0.08	0.89 ± 0.09	0.602(NS)
**BF (%)**	22.10 ± 7.81	17.59 ± 6.89	0.001	34.45 ± 7.96	26.70 ± 9.99	<0.001
**SBP (mm Hg)**	138 ± 25	142 ± 31	0.773(NS)	134 ± 29	129 ± 29.	0.525(NS)
**DBP (mm Hg)**	85 ± 18	88 ± 18	0.346(NS)	84 ± 16	81 ± 13	0.192(NS)
**RHR (bpm)**	74 ± 11	76 ± 13	0.217(NS)	78 ± 12	81 ± 12	0.408(NS)
**Blood glucose (mmol/l)**	5.15 ± 1.36	5.32 ± 2.89	0.973(NS)	5.24 ± 1.97	5.20 ± 2.11	0.057(NS)
**TC(mmol/l)**	4.44 ± 1.02	3.57 ± 0.97	<0.001	4.71 ± 1.04	3.14 ± 0.97	<0.001
**HDL-c (mmol/l)**	1.93 ± 0.89	2.60 ± 0.10	<0.001	2.10 ± 0.90	2.25 ± 0.91	0.323(NS)
**LDL-c (mmol/l)**	1.97 ± 1.14	1.44 ± 0.46	0.005	2.05 ± 1.21	1.36 ± 0.17	<0.001
**TG (mmol/l)**	1.23 ± 0.39	1.17 ± 0.64	0.017	1.27 ± 0.44	1.17 ± 0.40	0.066(NS)
**LDL/HDL-c Ratio**	1.64 ± 2.18	0.70 ± 0.58	0.001	1.59 ± 2.45	0.73 ± 0.36	0.009

Values are means ± SD; n, number of subjects

### Anthropometric characteristics

Table 1 shows gender specific means for anthropometric measurements in both urban and rural areas. Men were about 10 cm taller than women with significant differences between urban (1.64 ± 0.08cm; 95% Cl) and rural areas (1.62 ± 0.08cm; 95% Cl). WHR means did not differ between genders and study areas; but BMI and WC values were significantly higher in urban than in rural area (p<0.001), both in men and women. The BF% was also significantly higher (p ≤0.001) in urban than rural area, both in men and women.

**Figure 1:**
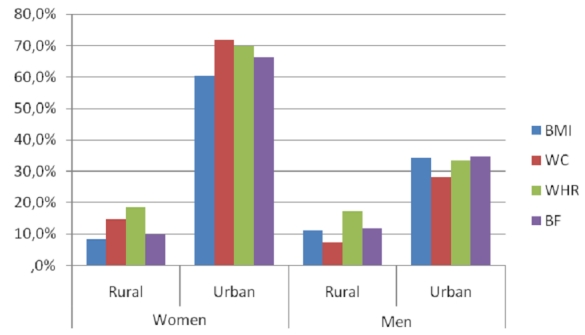
Prevalence of anthropometric risk factors respectively within the study population

**Figure 2:**
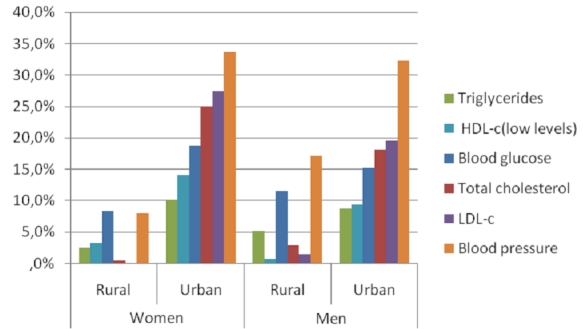
Prevalence of metabolic risk factors respectively within the study population

The prevalence rates of high BMI, WC and BF% were significantly higher in urban than in rural area in men (p=0.004, p=0.002 and p=0.004) and women (p<0.001) respectively. WHR showed the same tendency but the difference was not significant (Figure 1). High levels of TC, TG and LDL-c were significantly more prevalent in urban than in rural men (p=0.008, p=0.003 and p<0.001) and women (p<0.001, p=0.027 and p<0.001) respectively. The prevalence rate of hyperglycaemia was significantly higher in urban women compared to rural ones (p=0.013). In men, the tendency was similar but not significant. HDL-c's levels were lower in urban compared to rural area. This difference was significant men (p=0.015) but not in women. High blood pressure prevalence was higher in the urban than in the rural area but this was not significant (Figure 2).

### Metabolic characteristics

Table 1 shows gender specific means for metabolic measurements in both urban and rural areas. Age group differences are shown in Table 2 and Table 3 for both women and men respectively. Although the differences were not significant, SBP and DBP were higher in rural than in urban men except for the <40 years age group and higher in urban than in rural women. The highest values were found in rural men. The RHR was higher in rural than in urban women and men, and this difference was significant in the youngest women's age group (p=0.04). Blood glucose was higher in rural than in urban men, but lower in rural compared to urban women. The older age group showed an inverse tendency for the respective gender variations, and this was significant in women (p=0.030). TG were higher in urban than rural women but it was not significant. TG levels were significantly higher in urban than rural in men (p=0.017), especially in >55 years age group (p=0.004). Rural men had significantly higher HDL-c compared to urban ones (p<0.001), particularly in the oldest age group. HDL-c was higher in rural compared to urban women, but the difference was not significant. TC and LDL-c were significantly higher in urban than in rural area, both in men (p=0.005) and in women (p<0.001). 

**Table 2:** Metabolic profiles by age in urban and rural women Values are means ± Standard deviation

	< 40 years			40 – 55 years			>55 years		
	
	Urban	Rural	P	Urban	Rural	P	Urban	Rural	P
n	**62**	**12**		**94**	**26**		**70**	**23**	
**SBP**	118 ± 18	111 ± 13	0.194(NS)	133 ± 24	124 ± 13	0.180(NS)	148 ± 34	145 ± 24	0.827(NS)
**DBP**	78 ± 15	76 ± 8	0.849(NS)	84 ± 13	79 ± 11	0.150(NS)	90 ± 19	85 ± 17	0.332(NS)
**RHR (bpm)**	79 ± 14	88 ± 12	0.040	79 ± 11	79 ± 10	0.819(NS)	77 ± 13	78 ± 14	0.645(NS)
**Blood glucose (mmol/l)**	4.91 ± 1.74	4.04 ± 1.40	0.340(NS)	5.09 ± 1.28	5.04 ± 1.66	0.265(NS)	5.72 ± 2.72	6.00 ± 2.56	0.030
									
n	**56**	**9**		**81**	**22**		**61**	**18**	
**TC(mmol/l)**	4.37 ± 0.87	2.43 ± 0.98	<0.001	4.76 ± 1.00	3.11 ± 0.96	<0.001	4.97 ± 1.19	3.54 ± 0.78	<0.001
**HDL-cl (mmol/l)**	2.09 ± 1.04	2.56 ± 0.83	0.146(NS)	2.09 ± 0.91	2.25 ± 0.89	0.232(NS)	2.09 ± 1.04	2.56 ± 0.83	0.462(NS)
**LDL-cl (mmol/l)**	1.70 ± 1.03	1.35 ± 0.06	0.569(NS)	2.12 ± 1.14	1.35 ± 0.15	0.001	2.28 ± 1.38	1.39 ± 0.22	0.012
**TG (mmol/l)**	1.34 ± 0.33	1.30 ± 0.44	0.055(NS)	1.25 ± 0.48	1.13 ± 0.35	0.363(NS)	1.34 ± 0.33	1.30 ± 0.44	0.061(NS)
**LDL/HDL-c Ratio**	1.04 ± 1.02	1.01 ± 0.41	0.235	1.45 ± 1.35	0.72 ± 0.37	0.020	2.30 ± 3.95	0.60 ± 0.24	0.012

Values are means ± SD; n, number of subjects

**Table 3:** Metabolic profiles by age in urban and rural men

	<40 years			40 – 55 years			>55 years		
	
	Urban	Rural	P	Urban	Rural	P	Urban	Rural	P
n	**25**	**16**		**43**	**13**		**38**	**30**	
**SBP**	127 ±; 21	121 ±; 13	0.329(NS)	140 ±; 26	144 ±; 28	0.648(NS)	143 ±; 25	152 ±; 35	0.394(NS)
**DBP**	79 ±; 14	79 ±; 14	0.957(NS)	89 ±; 18	92 ±; 19	0.554(NS)	83 ±; 19	91 ±; 19	0.102(NS)
**RHR(bpm)**	74 ±; 10	82 ±; 13	0.073(NS)	74 ±; 12	72 ±; 8	0.580(NS)	73 ±; 12	75 ±; 15	0.361(NS)
**blood glucose (mmol/l)**	5.16 ±; 1.27	4.91 ±; 0.86	0.718(NS)	5.20 ±; 1.84	4.79 ±; 2.20	0.907(NS)	5.08 ±; 0.60	5.77 ±; 3.72	0.887(NS)
									
n	**21**	**10**		**37**	**12**		**32**	**27**	
**TC(mmol/l)**	4.44 ±; 1.14	3.28 ±; 1.36	0.028	4.67 ±; 0.98	3.73 ±; 0.95	0.012	4.18 ±; 0.96	3.60 ±; 0.81	0.021
**HDL-cl (mmol/l)**	1.98 ±; 0.88	2.34 ±; 1.19	0.660(NS)	2.12 ±; 0.97	2.55 ±; 1.25	0.609(NS)	1.67 ±; 0.75	2.71 ±; 0.79	<0.001
**LDL-cl (mmol/l)**	1.92 ±; 1.26	1.34 ±; 0.16	0.530(NS)	2.02 ±; 1.11	1.58 ±; 0.68	0.163(NS)	1.95 ±; 1.14	1.42 ±; 0.42	<0.018
**TG (mmol/l)**	1.24 ±; 0.42	1.32 ±; 0.74	0.583(NS)	1.17 ±; 0.40	1.33 ±; 0.96	0.329(NS)	1.27 ±; 0.43	1.05 ±; 0.38	0.004
**LDL/HDL-c Ratio**	1.50 ±; 1.60	0.71 ±; 0.37	0.428(NS)	1.68 ±; 2.81	0.88 ±; 0.87	0.236(NS)	1.68 ±; 1.65	0.61 ±; 0.48	<0.001

Values are means ±; SD; n, number of subjects

### Correlations

Table 4 and Table 5 show the correlations of the anthropometric parameters with the metabolic ones in women and men respectively. SBP was positively correlated to BMI in urban women, in both urban and rural men; and to WC and BF % in urban and rural women and men. DBP was positively correlated to BMI and WC in urban and rural women and men; and to BF % in urban and rural women, in urban men. The only correlation of TG was with BF % in urban men. Blood glucose positively correlated to BMI, WC and BF % in urban women and rural men. In urban women and men, we noted a positive correlation between TC and BMI as well as BF %. In the rural area, TC only correlated to WC in men. LDL-c was correlated to BMI in urban women and men, to WC in urban men and to BF % in urban women and men. After adjustment to age, WC remained correlated to SBP and DBP in rural men and women and in urban men. WC also correlated with TC and LDL-c in urban men. Correlation of BMI with DBP and TC persisted in rural and urban women respectively; as well as with SBP and TC in urban men. Blood glucose only correlated with WHR in urban women. BF% correlated with TC, TG and LDL-c. 

**Table 4:** Pearson's correlations coefficients of the anthropometric parameters with metabolic variables in women

	Age		BMI		WC		WHR		BF%	
	
	Urban	Rural	Urban	Rural	Urban	Rural	Urban	Rural	Urban	Rural
**SBP**	0.464^***^	0.505^***^	0.172^**^	0.145	0.237^***^	0.366^**^	0.283^***^	0.273^*^	0.312^***^	0.357^**^
**DBP**	0.265^***^	0.072	0.180^**^	0.287^*^	0.211^**^	0.416^**^	0.182^**^	0.121	0.254^***^	0.300^*^
**RHR**	−0.165^*^	−0.261^*^	0.008	0.027	−0.037	−0.018	−0.081	−0.147	−0.091	−0.160
**Blood glucose**	0.170^*^	0.376^**^	0.203^**^	0.029	0.198^**^	−0.100	0.179^**^	0.240	0.194^**^	0.140
**Total cholesterol**	0.195^**^	0.427^**^	0.169^*^	−0.039	0.187^**^	−0.014	0.116	0.440^**^	0.241^**^	0.117
**Triglycerides**	0.061	0.345^*^	−0.070	−0.022	0.013	0.042	0.062	0.224	−0.041	0.170
**LDL-c**	0.168^*^	−0.037	0.154^*^	0.153	0.122	0.019	−0.019	−0.091	0.150	0.223
**HDL-c**	−0.016	0.324^*^	−0.011	−0.059	0.019	−0.064	0.149^*^	0.344	0.046	0.023

***: p<0.001;

**: p<0.01;

*:p<0.05

**Table 5:** Pearson's correlations coefficients of the anthropometric parameters with metabolic variables in men

	Age		BMI		WC		WHR		BF%	
	
	Urban	Rural	Urban	Rural	Urban	Rural	Urban	Rural	Urban	Rural
**SBP**	0.222^*^	0.406^**^	0.269^**^	0.315^*^	0.351^***^	0.463^***^	0.242^*^	0.321^*^	0.354^***^	0.281^*^
**DBP**	0.005	0.228	0.298^**^	0.337^**^	0.350^***^	0.407^**^	0.213^*^	0.289^*^	0.265^**^	0.182
**RHR**	−0.187	−0.154	−0.009	0.156	0.014	0.194	0.107	0.224	0.080	0.089
**Blood glucose**	0.053	0.070	−0.014	0.316^*^	0.018	0.327^*^	0.075	0.056	0.043	0.341^*^
**Total cholestérol**	−0.176	0.137	0.327^**^	0.181	0.263^*^	0.290^*^	0.026	0.311^*^	**0.268** ^*^	0.098
**Triglycérides**	0.123	−0.091	0.102	0.085	0.106	0.177	0.013	0.148	**0.294** ^**^	0.126
**LDL-c**	0.034	0.038	0.337^**^	0.054	0.257^*^	−0.032	0.083	0.041	**0.322** ^**^	0.037
**HDL-c**	−0.246^*^	0.215	−0.109	0.070	−0.036	0.230	−0.045	0.273	−0.171	0.080

***=p<0.001;

**=p<0.01;

*=p<0.05

### Regression

In a multiple regression analysis, the impact of age, BMI, WC and BF% was evaluated with each metabolic variable. Age appeared to be an independent and significant predictor of SBP in this population (β=0.404, p <0.001), of DBP in rural men (β=3.055, p=0.004), of RHR in urban and rural men (β=-4.078, p <0.001) and of blood glucose in rural women (β=2.760, p=0.009). WC was an independent predictor of DBP and RHR in rural women (β=2.079, P=0.045) and men (β=3.041, p=0.004). BF % was significantly associated with DBP in rural men (β=-2.166, P=0.037), and RHR in rural and urban men (β=2.228, P=0.031). BMI was a significant and independent predictor of RHR only in rural men (β=-3.169, P=0.002). None of the predictors was related to TC, HDL-c and LDL-c concentrations.

### Diabetes in the population

We found 10 men and 20 women were diabetic, representing 6.6% of the whole, 8.3% and 6.0% of the rural and the urban populations respectively were diabetic. General and metabolic characteristics are shown in Table 6. Age and RHR were higher in diabetic than in non-diabetic subjects. This difference was significant in women (p=0.007; p=0.026 respectively) for age and RHR. BMI, WC and WHR were higher in subjects with diabetes than in those without diabetes, but this was not significant. Except for blood glucose, metabolic parameters did not differ significantly between diabetic and non-diabetic men and women. SBP, DBP and TG were higher in diabetic than non-diabetic subjects. This tendency was the same with HDL-c in women. TC and LDL-c did not differ between diabetic and non-diabetic women, and HDL-c varied similarly in men. TC and LDL-c were higher in subjects with diabetes than in those without diabetes. 

**Table 6:** General and metabolic characteristics in diabetic and non-diabetic subjects

	Men			Women		
		
	Diabetic	Non diabetic	p	Diabetic	Non diabetic	p
n	**10**	**154**		**20**	**268**	
**Age (years)**	57.6 ± 11.0	51.3 ± 15.8	0.222	57.8 ± 14.7	48.2 ± 14.1	0.007
**BMI (kg/cm^2^)**	25.9 ± 4.4	25.4 ± 4.8	0.744	28.9 ± 7.1	28.5 ± 6.2	0.610
**WC (cm)**	94.8 ± 10.6	89.4 ± 12.3	0.156	97.0 ± 14.4	93.7 ± 13.7	0.302
**WHR (cm)**	0.9 ± 0.1	0.9 ± 0.1	0.534	0.9 ± 0.08	0.9 ± 0.1	<0.001
**BF (%)**	21.4 ± 6.5	20.5 ± 7.9	0.587	35.8 ± 7.7	33 ± 9	0.129
**SBP (mm Hg)**	148 ± 37	139.6 ± 27.4	0.578	134.9 ± 22.9	133 ± 28	0.361
**DBP (mm Hg)**	93 ± 15	85.9 ± 18.6	0.137	89.4 ± 16.1	83 ± 16	0.069
**RHR (bpm)**	80 ± 10	74.8 ± 12.9	0.181	84.0 ± 7.2	79 ± 13	0.026
**Blood glucose (mmol/l)**	10.8 ± 4.8	4.9 ± 1.0	<0.001	10.7 ± 3.5	4.8 ± 1.0	<0.001
						
n	**9**	**129**		**17**	**231**	
**TC (mmol/l)**	3.7 ± 1.0	4.2 ± 1.1	0.206	4.4 ± 1.7	4.4 ± 1.2	0.774
**TG (mmol/l)**	2.5 ± 0.9	2.1 ± 1.0	0.226	2.2 ± 1.0	2.1 ± 0.9	0.717
**LDL-c (mmol/l)**	1.4 ± 0.3	1.8 ± 1.0	0.482	1.9 ± 1.4	1.9 ± 1.1	0.771
**HDL-c (mmol/l)**	1.2 ± 0.4	1.2 ± 0.5	0.817	1.4 ± 0.4	1.2 ± 0.4	0.131
**LDL/HDL c Ratio**	0.6 ± 0.2	1.4 ± 1.9	0.303	1.5 ± 2.1	1.4 ± 2.2	0.722

Values are means ± SD; n, number of subjects

## Discussion

It is clear that nutritional, demographic, epidemiological and socioeconomic transitions occurring in many developing countries are favourable to changes in nutritional and life style conditions [2,8]. In Cameroon, the differences between the modern and traditional way of living in the urban and rural areas greatly impact health conditions [11,23]. In the present study held in urban and rural Cameroon, the differences in metabolic profile between urban and rural people are indicated.

The prevalence rates of the anthropometric and the metabolic risk factors are higher in urban than in rural area. This is certainly in relation with the different lifestyles in the two areas, and it is in agreement with Sobngwi et al (2002) findings that physical inactivity was associated with obesity, hypertension and diabetes [23].

Fasting blood glucose was significantly higher in the oldest age group of rural women; this is consistent with the strong relation we found between age and blood glucose in rural women, but contrary to the results of Sobngwi et al in 2002 [23], who reported a significantly higher fasting glycaemia in urban Cameroonian women. The reason could be the age in the rural area, as hyperglycaemia and diabetes have been associated with age [24].

Total cholesterol was significantly higher in urban than in rural area in both genders and age categories. The difference between urban and rural areas regarding the feeding habits could have an impact on lipid metabolism. Urbanization usually involves varying degrees of modernization and westernization which have an impact on dietary habits. The urban environment entails important changes in lifestyles, economic activities, exposure to marketing and reference group influences. All these impinge on traditional diets and lead to shifts in food consumption patterns [25]. In their study concerning food consumption in developing country cities and modernisation, Delpeuch and Maire in 2004 [8] showed that hypercholesterolemia, diabetes and cardiovascular diseases in urban area were due to sedentary life and the nutritional transition characterized by increased consumption of foods rich in fat, rapid sugars and energy. Excess saturated fat inhibits the purification of cholesterol by the liver and thus increases cholesterol production by the same organ. As in our study, people living in the Cameroon rural area still have traditional diets with many vegetables and season fruits, products that facilitate cholesterol purification by the liver.

The higher level of HDL-c found in the rural area, although not significant except in the oldest women's age group, is inversely correlated to LDL-c whose level is significantly higher in the urban area. This is consistent with data found in the literature and confirms the low values of the LDL/HDL-c ratio, meaning that both lipoproteins are well metabolised. The different concentrations between the study areas may also be due to the difference in diets and physical activity of both areas, and could impact on the disease pattern although black women seem to be relatively protected against CVD [26]. Sone et al.[27] noted that in a Japanese population, low HDL-c level was not a significant risk factor for CVD, in contrast to the United Kingdom Prospective Diabetes Study in which HDL-c levels were significantly associated with CVD risk in a British Caucasian population.

The prevalence of diabetes in our study was 6.0% in the urban area. This is comparable to the prevalence obtained by CAMBOD in urban Cameroon in 2004 [17] and greater than that reported by Mbanya a decade before [10,18]. The prevalence of diabetes was higher in rural than in urban population; which is inconsistent with the results of many studies reported till date [10,18]. This may be due to the small size of our sample, especially in the rural population. However, these results are consistent with the fact that the lower prevalence of diabetes in Sub Saharan Africa compared to European countries could be explained by under diagnosis of the disease [28].

Age was higher in diabetic than non-diabetic subjects. Kengne and Awah found in 2008 that age was a potential determinant of death in rural Cameroon [29]. This age effect may be related to its contribution to cardiovascular diseases occurring, especially in the occurrence of type 2 diabetes [24]. Although not statistically significant, anthropometric parameters are higher in diabetic compared to non-diabetic subjects. This finding is consistent with the results of an international study which showed that BMI and particularly WC were both strongly linked to CVD and especially to type 2 diabetes [30]. However, the weakness of this relation is consistent with the absence of association between anthropometric parameters and blood glucose in this study. This may be explained by the small size of our sample. Blood pressure and resting heart rates were higher in diabetic than in non-diabetic subjects. It is known that elevated blood pressure has always been associated to glucose intolerance and hyperinsulinemia which are features of type 2 diabetes [30]. Lipid profile shows higher triglyceride levels in diabetic compared to non-diabetic subjects; this is commonly observed. Surprisingly, the other parameters of lipid profile are closer to the objectives in diabetic than in non-diabetic subjects. This discrepancy may be due to the different sample sizes in the studied groups.

## Conclusion

In conclusion, age appeared to be an independent predictor of SBP, DBP and RHR in men, especially in the rural area. Blood glucose was significantly higher in rural compared to urban women, especially in the >55 years age group and this in agreement with the higher rate of diabetes in the rural area. Despite correlations found between BMI, WC and BF% and blood glucose in urban and rural women, anthropometric parameters did not predict blood glucose levels in rural women, but age did. Except for HDL-c values, blood lipid levels were significantly higher in urban than in rural men and women, and the middle and the upper age groups were the most affected. BMI, WC and BF% correlated with total cholesterol and LDL-c in urban men and women. TC was associated with WC in rural men. HDL-c did not correlate with any of the anthropometric parameters. Age did not predict lipid profiles in this study. It would be very early with this study to make assertions or to draw conclusions. Many of our results remain unclear and justify the necessity to undertake more exhaustive studies in order to determine the responsibility of the various determinants on the studied metabolic variables.
